# Supplementary material for: The application of blockchain technology in data trading: a systematic review
Source: PeerJ Comput Sci. 2025 May 30;11:e2925. doi: 10.7717/peerj-cs.2925 (PMC12192792; doi:10.7717/peerj-cs.2925)
Supplement: Supplemental Information 1 [file peerj-cs-11-2925-s001.docx]

| **Section and Topic** | **Item #** | **Checklist item** | **Location where item is reported** |
| --- | --- | --- | --- |
| **TITLE** | | |  |
| Title | 1 | The report is a systematic review. | 1 |
| **ABSTRACT** | | |  |
| Abstract | 2 | The abstract includes a structured format with background, objectives, and key results. | 1 |
| **INTRODUCTION** | | |  |
| Rationale | 3 | Described in the introduction. | 1, 2 |
| Objectives | 4 | Stated in the introduction. | 3 |
| **METHODS** | | |  |
| Eligibility criteria | 5 | Stated in the methodology. | 4, 5 |
| Information sources | 6 | Described in the methodology. | 4, 5 |
| Search strategy | 7 | Stated in the methodology. | 4, 5 |
| Selection process | 8 | Stated in the methodology. | 4, 5 |
| Data collection process | 9 | Described in the methodology. | 4, 5 |
| Data items | 10a | Stated in the methodology. | 4, 5 |
|  | 10b | Stated in the methodology. | 4, 5 |
| Study risk of bias assessment | 11 | Described in the methodology. | 4, 5 |
| Effect measures | 12 | Described in the methodology. | 4, 5 |
| Synthesis methods | 13a | Stated in the methodology. | 4, 5 |
|  | 13b | Stated in the methodology. | 4, 5 |
|  | 13c | Described in the methodology. | 4, 5 |
|  | 13d | Stated in the methodology. | 4, 5 |
|  | 13e | Described in the methodology. | 4, 5 |
|  | 13f | Stated in the methodology. | 5, 6 |
| Reporting bias assessment | 14 | Stated in the methodology. | 5, 6 |
| Certainty assessment | 15 | Stated in the methodology. | 5, 6 |
| **RESULTS** | | |  |
| Study selection | 16a | Described in the exploratory analysis of results. | 6 |
|  | 16b | Described in the exploratory analysis of results. | 6 |
| Study characteristics | 17 | Stated in the exploratory analysis of results. | 7, 8 |
| Risk of bias in studies | 18 | Stated in the exploratory analysis of results. | 7, 8 |
| Results of individual studies | 19 | Stated in the exploratory analysis of results. | 7, 8 |
| Results of syntheses | 20a | Stated in the exploratory analysis of results. | 7, 8 |
|  | 20b | Described in the exploratory analysis of results. | 7, 8 |
|  | 20c | Described in the exploratory analysis of results. | 7, 8 |
|  | 20d | Stated in the exploratory analysis of results. | 8, 9 |
| Reporting biases | 21 | Described in the exploratory analysis of results. | 8, 9 |
| Certainty of evidence | 22 | Described in the exploratory analysis of results. | 8, 9 |
| **DISCUSSION** | | |  |
| Discussion | 23a | Described in the limitations and discussions. | 9 |
|  | 23b | Stated in the limitations and discussions. | 9 |
|  | 23c | Described in the limitations and discussions. | 10 |
|  | 23d | Stated in the limitations and discussions. | 10 |
| **OTHER INFORMATION** | | |  |
| Registration and protocol | 24a | Registration does not apply. |  |
|  | 24b | The protocol is described in the Methods. |  |
|  | 24c | No amendments. |  |
| Support | 25 | The funder had no role in study selection, data extraction, or manuscript preparation. No non-financial support was received. |  |
| Competing interests | 26 | No conflicts exist. |  |
| Availability of data, code and other materials | 27 | The materials are described in the Methods. |  |
